# Suggested Corrections to the Farm Family Exposure Study

**DOI:** 10.1289/ehp.114-a633a

**Published:** 2006-11

**Authors:** David T. Mage

**Affiliations:** Department of Public Health, Temple University (retired), Newark, Delaware, E-mail: magedonner@aol.com

Acquavella et al. (2004) reported glyphosate exposure analyses from the Farm Family Exposure Study (FFES) using biomonitoring. The authors “analyzed urine samples for creatinine to assess the completeness of daily samples,” but inadvertently used as “the normal range” 0.8–1.4 mg/dL and 0.5–1.1 mg/dL for males and females, respectively, which are the normal ranges of serum creatinine [National Institutes of Health (NIH) 2003]. The NIH normal values for urine creatinine are 24-hr total excretion values ranging from “500 mg/day to 2000 mg/day” (NIH 2006). Thus, Acquavella et al. (2004) needed to compare the 24-hr creatinine collection (urine creatinine concentration × urine volume) to each individual’s normative value of daily creatinine excretion based on age, sex, and body surface area (Cockcroft and Gault 1976).

Acquavella et al. (2004) also did not correct for the initial conditions. Of 47 farmers, 7 had 24-hr urinary glyphosate concentrations above the minimum detectable value of 1 ppb immediately before the start of their application. Such a farmer who had zero exposure during the monitored application would have excreted glyphosate over the following 4-day collection period in an amount estimable from the measured individual excretion rates. For a truly unexposed applicator to be shown to have a dosage statistically similar to zero, this estimated total 4-day excretion with zero exposure must be subtracted from the 4-day collection value.

In addition, Acquavella et al. (2004) evaluated one application per family and called it only a “potential limitation,” without realizing that this may vitiate their study. If all 47 FFES subjects with complete data had an identical exposure distribution, any single applicator sampled 47 different times would have an expectation of presenting exposure data with a statistically similar mean and variance as the FFES 47 sampled only once each. Therefore Acquavella et al. (2004) cannot reject the possibility that all 47 applicators have a similar exposure distribution by taking only one sample from each. This is because an applicator’s pesticide exposure is a stochastic process (accidents happen) that varies wildly from day to day, unlike the applicator’s weight that is a relatively constant process that barely varies from day to day. Therefore a single measured exposure provides no statistical information for estimating the applicator’s mean exposure over any time period other than the day measured. Furthermore, farmers’ pesticide exposures are not results of a stationary process, (defined as a time series in which the mean and variance of measured exposures, over a sufficiently long period from time 1 to time 2, are constants independent of choice for time 1). In an earlier study, we (Mage et al. 2000) successfully modeled the risk of accidental high pesticide exposure events in the Agricultural Health Study population as decreasing with the increasing lifetime number of application days. As one might expect, we showed that as an applicator gains experience, the risk of high exposure decreases. Therefore differences in lifetime experience of the FFES applicators prior to sampling introduce another variance component into the analysis.

In conclusion, Acquavella et al. (2004) treated a single sample at the end of a non-stationary time series—with declining mean and finite variance—as if it were actually the true mean value of a stationary process with zero variance. I recommend that Acquavella et al. (2004) consider revising their analyses by correcting properly for incomplete urine collection, correcting for the initial condition of prior glyphosate exposure, and adjusting for the experience of the applicator (lifetime number of application days) as an explanatory variable.

## References

[b1-ehp0114-a0633a] Acquavella JF, Alexander BH, Mandel JS, Gustin C, Baker B, Chapman P (2004). Glyphosate biomonitoring for farmers and their families: results from the Farm Family Exposure Study. Environ Health Perspect.

[b2-ehp0114-a0633a] Cockcroft DW, Gault MH (1976). Prediction of creatinine clearance from serum creatinine. Nephron.

[b3-ehp0114-a0633a] Mage DT, Alavanja MCR, Sandler DP, McDonnel CJ, Kross B, Rowland A (2000). A model for predicting the frequency of high pesticide exposure events in the Agricultural Health Study. Environ Res.

[b4-ehp0114-a0633a] NIH (National Institutes of Health) 2003. Creatinine - Serum. Available: http://www.nlm.nih.gov/medlineplus/ency/article/003475.htm [accessed 17 May 2006].

[b5-ehp0114-a0633a] NIH (National Institutes of Health) 2006. Creatinine - Urine. Available: http://www.nlm.nih.gov/medlineplus/ency/article/003610.htm [accessed 17 May 2006].

